# Charge-Complementary Polymersomes for Enhanced mRNA Delivery

**DOI:** 10.3390/pharmaceutics15122781

**Published:** 2023-12-15

**Authors:** HakSeon Kim, Yu-Rim Ahn, Minse Kim, Jaewon Choi, SoJin Shin, Hyun-Ouk Kim

**Affiliations:** 1 Division of Chemical Engineering and Bioengineering, College of Art, Culture and Engineering, Kangwon National University, Chuncheon-si 24341, Gangwon-do, Republic of Korea; gkrtjsv@kangwon.ac.kr (H.K.); reviana@kangwon.ac.kr (Y.-R.A.); minse0415@kangwon.ac.kr (M.K.); choijw8839@kangwon.ac.kr (J.C.); tlsthwls47@kangwon.ac.kr (S.S.); 2 Department of Smart Health Science and Technology, College of Art, Culture and Engineering, Kangwon National University, Chuncheon-si 24341, Gangwon-do, Republic of Korea

**Keywords:** ChargeSome, electrostatic interactions, endosomal escape, mRNA, polymersome, transfection

## Abstract

Messenger RNA (mRNA) therapies have emerged as potent and personalized alternatives to conventional DNA-based therapies. However, their therapeutic potential is frequently constrained by their molecular instability, susceptibility to degradation, and inefficient cellular delivery. This study presents the nanoparticle “ChargeSome” as a novel solution. ChargeSomes are designed to protect mRNAs from degradation by ribonucleases (RNases) and enable cell uptake, allowing mRNAs to reach the cytoplasm for protein expression via endosome escape. We evaluated the physicochemical properties of ChargeSomes using ^1^H nuclear magnetic resonance, Fourier-transform infrared, and dynamic light scattering. ChargeSomes formulated with a 9:1 ratio of mPEG-b-PLL to mPEG-b-PLL-SA demonstrated superior cell uptake and mRNA delivery efficiency. These ChargeSomes demonstrated minimal cytotoxicity in various in vitro structures, suggesting their potential safety for therapeutic applications. Inherent pH sensitivity enables precise mRNA release in acidic environments and structurally protects the encapsulated mRNA from external threats. Their design led to endosome rupture and efficient mRNA release into the cytoplasm by the proton sponge effect in acidic endosome environments. In conclusion, ChargeSomes have the potential to serve as effective secure mRNA delivery systems. Their combination of stability, protection, and delivery efficiency makes them promising tools for the advancement of mRNA-based therapeutics and vaccines.

## 1. Introduction

Owing to its unique advantages, messenger RNA (mRNA) is emerging as a potential therapeutic modality, offering a viable alternative to treatments based on deoxyribonucleic acid (DNA) [1,2]. mRNA facilitates the precise control of protein expression, making it a compelling choice for tailored therapies whereby targeted proteins may be generated as required [3]. In addition, once delivered to the intended cells, mRNA allows customization of the therapeutic effect by modifying the dosage and frequency, thereby offering therapeutic versatility [4]. Cells have the capacity to create the desired protein for a limited period, thereby reducing the possible dangers of excessive expression and the resulting negative consequences [5]. The use of mRNA as a therapeutic tool is promising; however, there are various obstacles to its use as a therapeutic agent [6]. One of the main obstacles is the inherent instability of mRNA molecules and their susceptibility to degradation. The presence of ribonucleases (RNases) in the extracellular milieu and body fluids may result in the rapid deterioration of mRNA molecules [7,8]. Furthermore, the restricted absorption rate of negatively charged and relatively large naked mRNA molecules is attributed to electrostatic repulsion of the cell membrane, which limits their therapeutic potential [7,9,10]. Therefore, a persistent problem in the field of mRNA delivery is the attainment of effective mRNA transportation to specific cells and tissues while avoiding degradation and unwanted immunological reactions [11]. To address these issues, studies are currently engaged in extensive investigations on diverse delivery methods, such as lipid- and polymer-based carriers, as shown by prior studies [12,13].

mRNA transporters must be proficient in two primary roles. Both internal and extracellular mRNA must be protected from ribonuclease-mediated degradation, and mRNA must also avoid endosomes and minimize side effects during cellular internalization. Lipid- and polymer-based carriers may improve the stability of intracellular mRNA transport [14,15]. It has been proposed that lipid- and polymer-based carriers play a pivotal role in facilitating the intracellular transport of mRNA into the cytoplasm, which is a critical step for enhancing the stability of mRNA delivery systems, as discussed in previous studies [16,17]. It has been proposed that lipid- and polymer-based carriers play a pivotal role in facilitating the intracellular transport of mRNA into the cytoplasm, which is a critical step for enhancing the stability of mRNA delivery systems, as discussed in previous studies [18]. Cationic lipids are used in lipoplex nucleotide delivery [19]. Electrostatic interactions of the delivery system with the negatively charged phosphate backbone of the mRNA facilitate the transportation of mRNA to the intended recipient cells, ensuring effective delivery. This process involves the attraction between the positively charged components of the delivery system and the negatively charged phosphate groups in the mRNA, allowing for the successful transport of mRNA to the desired cells [20]. mRNA vaccines depend on lipid-based carriers to deliver mRNA to the desired cells. Polymer-based carriers are chemically diverse, allowing fine control over release processes and dispersion patterns. Polymers with both ionic and cationic properties facilitate electrostatic interactions with nucleic acids, thereby significantly enhancing mRNA transport efficiency [21,22]. The presence of the polymer inside acts as a protective barrier, preventing the degradation of mRNA and ensuring a sustained release over an extended period. Therefore, toxicity and immunological responses are reduced [23,24]. This mRNA delivery strategy has promising potential for applications in gene therapy and vaccine production [25]. Intracellular mRNA-based drugs and vaccine delivery vehicles that utilize electrostatic attraction have been the subject of study. “Intracellular mRNA-based drugs” refer to therapeutic agents that rely on messenger RNA (mRNA) for intracellular delivery and gene expression modulation. These drugs are designed to enter target cells, deliver mRNA payloads to the cells’ interior, and induce specific cellular responses through mRNA translation. This broad category of therapies includes gene therapy and mRNA vaccines, leveraging the cell’s protein synthesis machinery for therapeutic purposes [26,27]. These systems generate particles through ionic interactions. Ionic interactions cause charged polymers to spontaneously self-assemble [15]. Electrostatic interactions stabilize nanoparticles under physiological conditions [28]. Electrostatic interactions between positively and negatively charged substances help nanoparticles encapsulate mRNA [29]. Electrostatic interactions wrap the mRNA in a core–shell structure, protecting it from enzyme degradation and other threats to its stability and preventing premature degradation. Ionic nanoparticles are stable at neutral pH. Disintegration releases mRNA in acidic environments such as endosomes or tumor microenvironments. This release method avoids endosomes and allows the mRNA molecules to reach the cytoplasm of the target cell [30]. A small positive charge on the cell surface may facilitate endosomal escape by promoting cell membrane interaction [31].

**Scheme 1:**
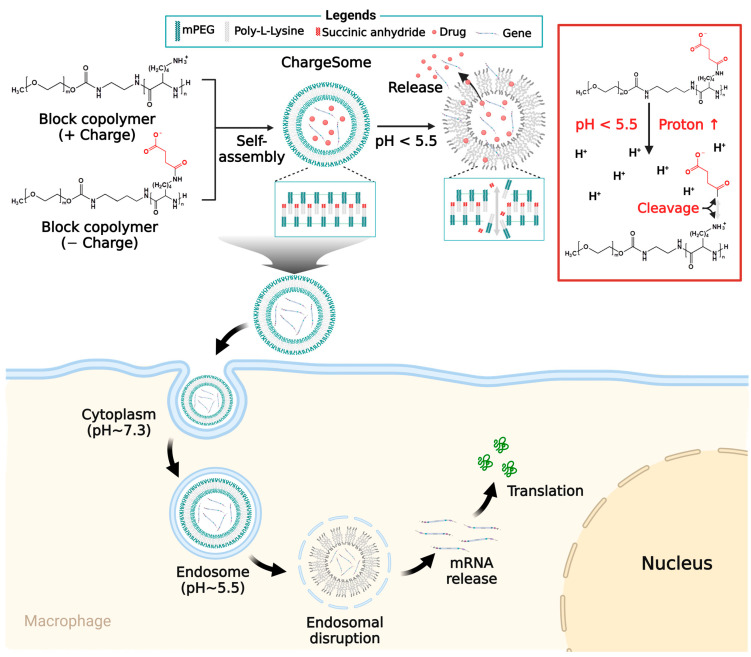
ChargeSome nanoparticles are created using a charged polymer. These nanoparticles are pH-sensitive, enabling them to disintegrate at a pH of 5.5 or lower, leading to the release of encapsulated genes. Upon cell entry, these nanoparticles are taken up in the form of endosomes. Once inside the endosomes with a pH below 5.5, the nanoparticles disintegrate, causing destabilization of the endosome membrane and facilitating their release into the cytoplasm through endosomal escape. The mechanism of this process is depicted in a scheme illustrating particle behavior within a cell.

In this study, we introduce the term “ChargeSomes” to describe pH-sensitive nanoparticles [32], demonstrating several notable advantages when compared to lipid-based carriers, particularly in terms of controlled mRNA release, responsiveness to the acidic endosomal environment, mRNA stability maintenance, cytotoxicity reduction, and mRNA protection. One approach to maintaining stability involves the use of cationic methoxy polyethylene glycol-block-poly-L-lysine (mPEG-b-PLL) and negatively charged methoxy polyethylene glycol-block-poly L-lysine-succinic anhydride (mPEG-b-PLL-g-SA), which effectively neutralize the charge and avoid interaction with RNase throughout the delivery process. Another benefit is its ability to be released in response to changes in pH [33,34]. Hydrolysis of the amide link between succinic anhydride and lysine in mPEG-b-PLL-SA occurs in an acidic environment at a pH of 5.5, resulting in the effective release of mRNA [35,36,37](Scheme 1). ChargeSomes are composed of polyethylene glycol (PEG), a polymer known for its biocompatibility [38]. This phenomenon decreases the likelihood of immune recognition and prolongs the circulation period, thereby enhancing safety and mitigating possible adverse consequences [39]. ChargeSomes were generated by electrostatic interactions between the cationic polymer mPEG-b-PLL and the anionic polymer mPEG-b-PLL-SA. By varying the proportions of mPEG-b-PLL and mPEG-b-PLL-SA, the strength of the electrostatic connections was effectively enhanced [37]. Furthermore, the ability to manipulate particle size allowed us to control crucial factors, including drug release and cellular absorption, thereby enhancing the overall effectiveness of the delivery system. This phenomenon plays a crucial role in sustaining the integrity and stability of messenger ribonucleic acids (mRNAs), thereby augmenting the efficacy of treatments that rely on mRNA [13]. To evaluate the immune response, cytokine production, and intracellular fate of pH-sensitive nanoparticles loaded with mRNA, we used RAW 264.7 cells, which can provide a practical model system for mRNA vaccine delivery experiments and contribute to a more comprehensive assessment of the potential of vaccine delivery systems. Nuclear magnetic resonance (NMR) and Fourier-transform infrared (FT-IR) spectroscopy were used to validate the chemical characterization of the copolymer. Dynamic light scattering (DLS), transmission electron microscopy (TEM), and zeta-potential tests were performed to determine the physicochemical characteristics of the particles and evaluate their potential as mRNA carriers. These nanoplatforms have potential applications as delivery systems for mRNA-based therapeutics and vaccines that use electrostatic attraction to facilitate successful intracellular administration.

## 2. Materials and Methods

### 2.1. Materials

H-lys(Z)-OH ≥ 99.0% (NT), triphosgene, tetrahydrofuran anhydrous (stabilized with BHT), N-hexane, ethyl acetate, and methoxy-poly(ethylene glycol)-NH2 (mPEG-NH2, MW 2000) were purchased from Laysan Bio, *N*,*N*-dimethylformamide (DMF) anhydrous, diethyl ether, trifluoroacetic acid, HBr/acetic acid solution (33%), succinic anhydride ≥ 99% (GC), dialysis tubing (molecular weight cutoff; MWCO 3.5 K), and Vivaspin 500 (300 KDa) were purchased from Sigma-Aldrich (Burlington, MA, USA), OVA–FITC was purchased from Thermo Fisher Scientific (San Jose, CA, USA), Neogreen was obtained from NEO Science (Dubai, United Arab Emirates), and CleanCap Enhanced Green Fluorescent Protein (EGFP) mRNA (5moU) was obtained from TriLink (San Diego, CA, USA).

#### Cells

The RAW 264.7 cell line was purchased from the Korean Cell Line Bank (KLCB 40071). RAW 264.7 cells were cultured in Dulbecco’s modified Eagle’s medium (DMEM) supplemented with 10% fetal bovine serum (FBS) and 1% antibiotic–antimycotic. Cell culture medium and supplements were obtained from Thermo Fisher Scientific (San Jose, CA, USA).

### 2.2. Synthesis of mPEG-b-PLL-SA

#### 2.2.1. Synthesis of Nε-Carbobenzoxy-L-lysine *N*-Carboxyanhydride (Lys(Z)-NCA)

Copolymerization was conducted in a fume hood. Lys(Z)-NCA was synthesized using the Fuchs–Farthing method [40,41,42]. To initiate the process, H-Lys(Z)-OH (3.5 g, 12.49 mmol) was added to a three-necked round-bottomed flask. Following the vacuum step, anhydrous THF (60 mL) was added under dry nitrogen atmosphere. Separately, triphosgene (1.68 g, 5.68 mmol) was dissolved in anhydrous THF (25 mL) in another flask and subsequently added to the Lys(Z)-THF suspension. The mixture was stirred at 40 °C for a duration of 3 h under a nitrogen atmosphere. Over time, the suspension gradually transitioned into a transparent state. After cooling to 25 °C, the solution was passed through a hydrophobic syringe filter. Subsequently, it was washed three times with cold n-hexane (300 mL) and separated under reduced pressure. The resulting material was dried to yield a white solid—Lys(Z)-NCA. The yield of Lys (Z)-NCA was 68.7%.

#### 2.2.2. Synthesis of Methoxy-poly(ethylene glycol)-block-poly(ε-Cbz-L-lysine) (mPEG-b-PLL(Z))

In the synthesis of mPEG-b-PLL(Z), Lys(Z)-NCA (3.50 g, 11.42 mmol) was added to a three-necked round-bottomed flask. After creating a sufficient vacuum, anhydrous DMF (60 mL) was introduced under a dry nitrogen atmosphere and dissolved by stirring. Separately, mPEG-NH2 (0.801 g, 0.38 mmol) was dissolved by adding anhydrous DMF (20 mL) and then introduced into the Lys(Z)-NCA solution. The mixture was stirred at 40 °C for a duration of 48 h under a nitrogen atmosphere, resulting in a vivid yellow color change. After cooling to room temperature, the solution was precipitated with cold diethyl ether (400 mL), followed by three rounds of purification under reduced pressure. The final product obtained was 2.56 g of a white solid referred to as mPEG-b-PLL(Z). The yield of mPEG-b-PLL (Z) was approximately 58%.

#### 2.2.3. Synthesis of Methoxy-poly(ethylene glycol)-block-poly(L-lysine) (mPEG-b-PLL)

To protect mPEG-b-PLL(Z), mPEG-b-PLL(Z) (3.42 g, 0.34 mmol) was dissolved in trifluoroacetic acid (34 mL). Subsequently, 6 mL of a HBr–acetic acid solution (33%) was added. The reaction was conducted in an ice bath and, after stirring for 2 h, the product was precipitated in cold diethyl ether. The obtained mPEG-b-PLL was dissolved in DMF. The mPEG-b-PLL solution was dialyzed using a membrane with an MWCO of 3.5 K. Purified mPEG-b-PLL was preserved after freeze-drying.

#### 2.2.4. Synthesis of mPEG-b-PLL-SA

mPEG-b-PLL-SA was synthesized via an amine reaction between mPEG-b-PLL and succinic anhydride (SA) [43]. Initially, mPEG-b-PLL (0.5 g, 0.084 mmol) was dissolved in distilled water at pH 8.5. Succinic anhydride was separately dissolved in dimethyl sulfoxide, and the two solutions were combined. The pH was adjusted to 8.0. After stirring for 24 h, the mPEG-b-PLL-SA solution was dialyzed for 3 days using a membrane with an MWCO of 3.5 K. The solution was then filtered using a syringe filter with a cellulose acetate membrane and subsequently freeze-dried to obtain mPEG-b-PLL-SA.

#### 2.2.5. Characteristics of Copolymer

The molecular structures of the synthesized Lys(Z)-NCA, mPEG-b-PLL(Z), mPEG-b-PLL, and mPEG-b-PLL-SA were characterized using ^1^H NMR spectrum recorded on a 600 MHz NMR spectrometer (Bruker, Bremen, Germany). The functional group modifications and chemical structures were analyzed using FT-IR spectroscopy [41].

### 2.3. Preparation and Characteristics of ChargeSomes

mPEG-b-PLL and mPEG-b-PLL-SA were dissolved in phosphate-buffered saline (PBS) at pH 7.4 and mixed in weight ratios of 1:9, 3:7, 5:5, 7:3, and 9:1 (3.3 mg/mL, total weight of both polymers). The solution was vortexed for 2 min and sonicated for 10 s. Subsequently, particles were formed by stirring for 1 h [44]. The size distribution of ChargeSomes was determined using DLS, and zeta-potential analysis was performed. Their size and morphology were characterized using TEM. Ovalbumin (OVA)-FITC encapsulation was achieved by adding OVA–FITC (8.89 µM) to the ChargeSome solution (3.3 mg/mL) and stirring the mixture for 12 h. Subsequently, free OVA–FITC was removed by centrifugation at 4700 g using a Vivaspin 500 filter (300 KDa). EGFP mRNA (5 moU) purchased from Trilink and was encapsulated during the ChargeSome formation process, followed by 2 min of vortexing. This process ensured that EGFP mRNA was evenly incorporated into the ChargeSome particles during their formation.

### 2.4. Stability and pH Reactivity of ChargeSomes

The stability and hydrodynamic diameter changes of ChargeSomes were assessed by dynamic light scattering (DLS). Diameter changes were monitored in PBS (pH 7.4) at weekly intervals for one month and then biweekly for confirmation. To investigate particle reactivity at a low pH (pH 5.0), diameter changes were measured in PBS at various time points (0 h, 1 h, 3 h, 7 h, 15 h, 1 d, 2 d, and 3 d) using DLS.

### 2.5. Cell Viability

The cytotoxicity of ChargeSomes formed with various mPEG-b-PLL:mPEG-b-PLL-SA ratios (1:9, 3:7, 5:5, 7:3, and 9:1) was assessed using RAW 264.7 cells. Cells (1 × 10^4^ RAW 264.7 cells per well) were cultured in sterile 96-well plates. Each well was filled with 200 μL of DMEM supplemented with 10% FBS and 1% antibiotic–antifungal and incubated at 37 °C with 5% CO_2_ for 24 h. Cell viability was assessed by adding 20 μL of ChargeSomes at various concentrations (0.05 mM, 0.10 mM, 0.21 mM, and 0.42 mM) after the incubation period. Similarly, the cell viabilities of ChargeSomes (1:9, 3:7, 5:5, 7:3, and 9:1) and lipofectamine were determined under optimal concentration conditions. Cell viability was evaluated using EZ-Cytox after a 12- and 24-h incubation period at 37 °C with 5% CO_2_ following the introduction of particles. Measurements were performed at 450 nm using a microplate reader.

### 2.6. In Vitro Analysis of OVA Cell Uptake

RAW 264.7 cells (5 × 10^4^) were seeded into confocal dishes for confocal laser-scanning microscopy (CLSM) measurement and incubated at 37 °C for 24 h in an atmosphere containing 5% CO_2_. Subsequently, the cells were treated with OVA–FITC-encapsulated ChargeSome and OVA–FITC solutions and cultured for 6 h (37 °C, 5% CO_2_). After the 6-h incubation, cells were washed with DPBS. Cell absorption of ChargeSomes was visualized using a laser-scanning microscope (Carl Zeiss LSM880 with Airyscan). Cells were stained with Hoechst to visualize the nucleus, and lysosomes were visualized by staining with Lysosome Red DND-99. For flow cytometry analysis, RAW 264.7 cells (5 × 10^5^) were dispensed into 6-well plates and cultured in an atmosphere containing 5% CO_2_ at 37 °C for 24 h. After the incubation, OVA–FITC-encapsulated ChargeSome and OVA–FITC solutions were added and incubated with 5% CO_2_ at 37 °C for 6 h. Following treatment, the cells were washed twice with DPBS and collected in 1 mL of phenol-free DMEM. Flow cytometry was performed using an LSR II flow cytometer (FACSymphony, Becton Dickinson, Franklin Lakes, NJ, USA), and the data were analyzed using FlowJo software version 10.9.0 (Tree Star, Inc., Ashland, OR, USA) [45]. To assess endosomal escape, cells were prepared in the same manner. Subsequently, OVA–FITC-encapsulated ChargeSomes were administered for 1, 2, 4, or 6 h. The uptake of OVA–FITC-encapsulated polymersomes and the dye was observed using CLSM, with subsequent staining of the nucleus and lysosomes after each hour. The images were analyzed using the ZENblue software version 3.4.

### 2.7. Gel Electrophoresis

For the gel retardation assay, 1.00% (*w*/*v*) agarose gel was prepared and used for gel electrophoresis. This experiment sought to assess the encapsulation of mRNA across different weight ratios and analyze the stability of the mRNA-encapsulated ChargeSomes [46]. The encapsulation of mRNA was confirmed for ChargeSome concentrations of 0.11, 0.21, 0.42, 0.84, and 1.68 mM, each with mRNA at a concentration of 8 µM. The samples were mixed with blue RNA-loading dye, loaded onto an agarose gel, and run for 40 min at 100 V in MOPS buffer. The stability of ChargeSomes and mRNA-encapsulated ChargeSomes was assessed in three different conditions: PBS, FBS, and RNase [47]. Naked mRNA was subjected to the same stability analysis for comparison. For each sample, 5 μL of ChargeSomes was mixed with 5 μL of one of the solvents (PBS, FBS, or RNase) and incubated at room temperature for 30 min. The subsequent procedure was conducted in the same manner as that for mRNA-encapsulated ChargeSomes according to the weight ratio. This analysis helped determine the stability of the mRNA-encapsulated particles in different environments.

### 2.8. In Vitro EGFP mRNA Transfection and Analysis of EGFP Translation Efficiency

For CLSM measurements, RAW 264.7 cells (5 × 10^4^) were seeded in confocal dishes and precultured in complete medium (DMEM) at 37 °C for 24 h in a 5% CO_2_ atmosphere. After 24 h, the medium was replaced with Opti-MEM (500 µL). EGFP mRNA-encapsulated ChargeSome at different ratios (1:9, 3:7, 5:5, 7:3, and 9:1) and naked mRNA solutions were applied to the cells and further cultured for 24 h at 37 °C in a 5% CO_2_ atmosphere. After approximately 8 h of culture, the medium was replaced with complete medium (DMEM), and the cells were incubated for an additional 24 h. Finally, the medium was replaced with phenol red-free medium. The cells were stained with Hoechst, and EGFP expression was visualized using confocal laser-scanning microscopy (CLSM). Additionally, RAW 264.7 cells were cultured under the same conditions as the cell uptake experiment for flow cytometry. After 24 h, the cell culture medium was replaced with Opti-MEM, and EGFP mRNA-encapsulated ChargeSome at different ratios (1:9, 3:7, 5:5, 7:3, and 9:1), and naked mRNA solutions were added and incubated at 37 °C for 24 h in a 5% CO_2_ atmosphere. The cells were then washed and collected in DPBS. Flow cytometry was performed using the LSR II flow cytometer (FACSymphony, Becton Dickinson, Franklin Lakes, NJ, USA), and the data were analyzed using FlowJo software.

## 3. Results and Discussion

### 3.1. Synthesis and Characterization of mPEG-b-PLL and mPEG-b-PLL-SA Copolymers for Efficient mRNA Delivery via ChargeSomes

To develop polymer-based delivery vehicles for mRNA delivery to immune cells, we synthesized the biodegradable and biocompatible copolymers mPEG-b-PLL and mPEG-b-PLL-SA. The copolymerization process was initiated by neighboring amino groups inside the mPEG-b-PLL structure, leading to the formation of the matching anhydride. The ring-opening polymerization approach was successfully employed for the efficient synthesis of mPEG-b-PLL(Z). This synthesis involved the use of lys(Z)-NCA as the monomer and mPEG-NH2 as the macroinitiator. Subsequently, mPEG-b-PLL was synthesized by removing the benzyloxycarbonyl group from mPEG-b-PLL(Z), and mPEG-b-PLL-SA was synthesized by reaction with succinic anhydride. The chemical structures of the intermediates and end products were thoroughly verified using ^1^H NMR and FT-IR spectroscopy, as depicted in Figures S1 and S2. The synthesis process was validated with respect to the Lys(Z)-NCA block by analyzing the NMR spectra [41]. All the peaks in the spectrum were distinctly assigned, indicating successful synthesis (Figure S1). The synthesis of mPEG-b-PLL(Z) was confirmed to be consistent, with characteristic peaks observed at 3.24, 4.10–4.30, 4.96, and 7.31 ppm [48]. Furthermore, deprotection of mPEG-b-PLL(Z) was verified by the disappearance of specific peaks at 5.12 and 7.25 ppm, indicating successful deprotection [48]. Subsequently, the mPEG-b-PLL-SA synthesis was verified by the appearance of a distinct peak at 2.5 ppm [49]. In Figure S2, the FT-IR data depicted distinct peaks [41]. Notably, Lys(Z)-NCA displayed characteristic peaks at wavenumber 1652 cm^−1^ (attributed to the C=O stretching vibration) and additional peaks at 1851, 1809, and 1776 cm^−1^, which corresponded to the O=C–O–C=O functional groups. Concurrently, mPEG-b-PLL(Z) exhibited peaks at wavenumbers 1692 and 1627 cm^−1^, confirming its presence in the sample. Furthermore, mPEG-b-PLL demonstrated its copolymeric nature by revealing peaks at 1650 (amide I) and 1520 cm^−1^ (amide II). Therefore, we successfully synthesized mPEG-b-PLL(Z) and mPEG-b-PLL-SA copolymers to form ChargeSomes for mRNA delivery.

### 3.2. Characterization and Stability Analysis of ChargeSomes at Different mPEG-b-PLL to mPEG-b-PLL-SA Ratios

**Figure 1:**
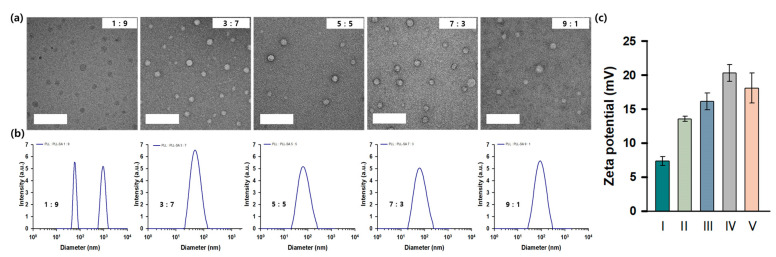
Morphological analysis and composition ratios of ChargeSomes at various ratios. (**a**) ChargeSomes with varying mPEG-b-PLL to mPEG-b-PLL-SA ratios (1:9, 3:7, 5:5, 7:3, and 9:1) were analyzed using TEM and negative staining. The aforementioned ratios serve as indicators of the hierarchical organization and interrelationships among the various components of ChargeSomes. The scale bar corresponds to 200 nm. (**b**) Size distribution of ChargeSomes determined by DLS. (**c**) Zeta-potential analysis of ChargeSomes using a zeta-potential particle size analyzer. I, II, III, IV, and V represent the zeta potential of the respective formation ratios of ChargeSomes (1:9, 3:7, 5:5, 7:3, and 9:1).

The subsequent stage entailed the characterization of ChargeSomes, which were chosen from a subset of mPEG-b-PLL/mPEG-b-PLL-SA to examine particle production in relation to the copolymer ratios. Seven unique fractions (0:10, 1:9, 3:7, 5:5, 7:3, 9:1, and 10:0) were analyzed. TEM images of the partially fabricated ChargeSomes showed spherical particles with bilayer structures at various ratios (1:9, 3:7, 5:5, 7:3, and 9:1), all of which had diameters smaller than 100 nm (Figure 1a). Interestingly, extreme ratios of 0:10 and 10:0, consisting solely of mPEG-b-PLL and mPEG-b-PLL-SA, respectively, did not yield spherical particles (Figure S3). To characterize the different ratios of mPEG-b-PLL and mPEG-b-PLL-SA comprising ChargeSomes, their sizes and surface charges were analyzed using dynamic light scattering (DLS) and zeta-potential measurements. For all ChargeSome samples, except the 1:9 ratio, the size of ChargeSomes indicated a low polydispersity index. The sizes observed for each ratio are shown in Figure 1b. Depending on the ratio of mPEG-b-PLL to mPEG-b-PLL-SA, the size increases to 39.54 and 704.94 nm at 1:9, 53.83 nm at 3:7, 78.85 nm at 5:5, 76.53 nm at 7:3, and 105.03 nm at 9:1. According to these results, ChargeSomes increased as the proportion of mPEG-b-PLL increased. Next, to determine the surface charge of ChargeSomes, zeta-potential measurements were performed (Figure 1c). The zeta potential was determined to be 7.37 at 1:9, 13.57 at 3:7, 16.13 at 5:5, 20.33 at 7:3, and 18.1 at 9:1. The zeta-potential value exhibited an increase as the ratio of mPEG-b-PLL increased. This indicates that the surface charge increased as the ratio of positively charged mPEG-b-PLL increased. Furthermore, the zeta-potential values, when analyzed in relation to the ratio of mPEG-b-PLL and mPEG-b-PLL-SA, indicated that the mPEG-b-PLL had a greater positive charge compared to the negative charge of mPEG-b-PLL-SA. The size distribution by DLS was identified as two peaks when the ratio of the ChargeSome component was 1:9 (mPEG-b-PLL: mPEG-b-PLL-SA). This is the result of a decrease in dispersibility, and it was confirmed that ChargeSomes were formed through TEM. These results confirmed the successful formation of ChargeSomes at various ratios (1:9, 3:7, 5:5, 7:3, and 9:1) via electrostatic interactions. The stability of ChargeSomes was evaluated at pH conditions of 5.0 and 7.4, as illustrated in Figure S5a. Over a six-week period at pH 7.4, ChargeSomes exhibited consistent stability. However, at pH 5.0, significant size changes were observed between 3 and 8 h, indicating the degradation of ChargeSomes. Hence, ChargeSomes exhibited pH-responsive behavior (Figure S5b). These observations indicate that ChargeSomes maintain structural stability under neutral pH conditions. However, when exposed to a low-pH environment, the particle morphology changes due to the inherent polymeric nature of ChargeSomes [35,37]. This provides fundamental insight into the pH response behavior of ChargeSomes.

### 3.3. Cell Viability Assays of ChargeSomes In Vitro

**Figure 2:**
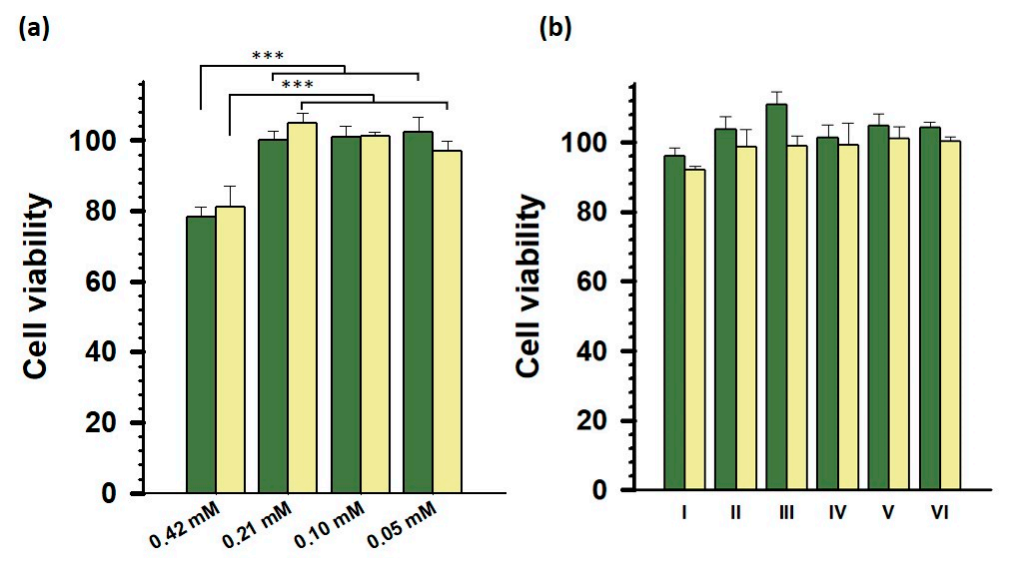
Effects of ChargeSomes on RAW 264.7 cell viability. (**a**) The cell viability of RAW 264.7 cells at 12 h (green), and 24 h (yellow) post-treatment with ChargeSome particles at various concentrations was evaluated using the EZ-Cytox assay. Student’s *t*-test and one-way ANOVA were used for statistical analysis. Data are presented as means ± S.D. (n = 5; *** *p* < 0.001). (**b**) RAW 264.7 cells underwent treatment with lipofectamine and distinct fractions of ChargeSome particles to enable a comparison of cell viability. I, II, III, IV, and V correspond to the mPEG-b-PLL/mPEG-b-PLL-SA ratios of 1:9, 3:7, 5:5, 7:3, and 9:1, respectively, while VI denotes lipofectamine.

Determining the safety of mRNA vaccine delivery systems, such as ChargeSomes, and in particular, their cytotoxicity while interacting with living cells, is critical for the evaluation process. Our previous synthesis and stability studies demonstrated the promising properties of ChargeSomes. Their biocompatibilities were determined in vitro. To evaluate the cytotoxicity of ChargeSomes, cell viability was measured using EZ-Cytox assay. High cell viability was observed at various concentrations, confirming the low cytotoxicity of ChargeSomes at the 7:3 ratio. Statistical significance was demonstrated with a *p*-value. However, when the concentration of ChargeSomes was changed between 0.21 mM (1.65 mg/mL) and 0.05 mM (0.41 mg/mL), no discernible difference in cell survival was seen. Cell viability was very slightly reduced to 78.4% at 12 h and 81.3% at 24 h when this dose was increased to 0.42 mM (3.3 mg/mL), as shown in Figure 2a. This result indicates that the optimal concentration for further in vitro experiments using ChargeSomes was 0.21 mM. The cytotoxicity of various ratios of mPEG-b-PLL and mPEG-b-PLL-SA was evaluated using ChargeSomes. Surprisingly, the cell viability remained constant across all ratios, as seen in Figure 2b. Compared to lipofectamine, the component ratio of ChargeSomes had no effect on cell viability. The stability of cell viability at different concentrations and compositions of ChargeSomes is important for safe mRNA delivery. The fact that cell viability barely changed until the concentration reached 0.42 mM indicates its suitability for a vaccine delivery platform [50]. These results suggest that consistent cell viability at different ChargeSome composition ratios reflects the absence of toxicity associated with ChargeSomes, indicating that cells are unlikely to be damaged by variations in ChargeSome composition.

### 3.4. OVA–FITC Uptake of ChargeSomes In Vitro

**Figure 3:**
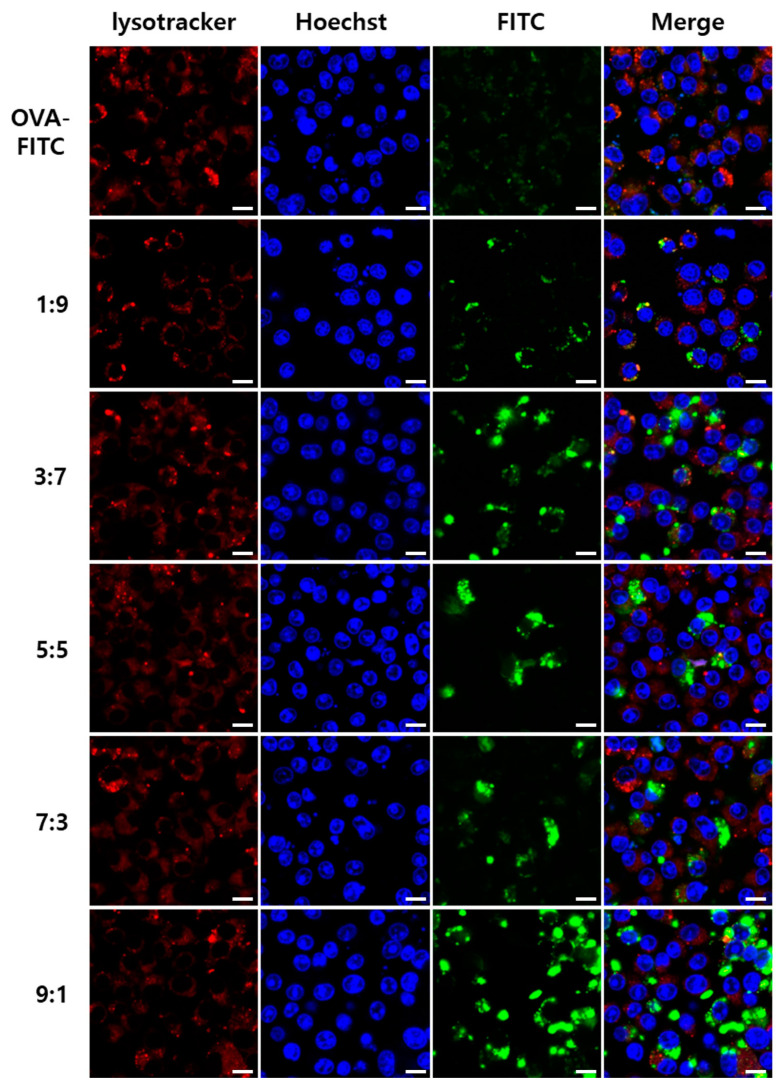
Cell uptake of OVA–FITC-encapsulated ChargeSomes in RAW 264.7 cells observed via CLSM. RAW 264.7 cells were incubated with OVA–FITC-encapsulated ChargeSomes (green) for 6 h, and lysosomes and nuclei were stained using Lysotracker-DND-99 (red) and Hoechst 33342 (blue), respectively. Scale bars represent 10 um.

**Figure 4:**
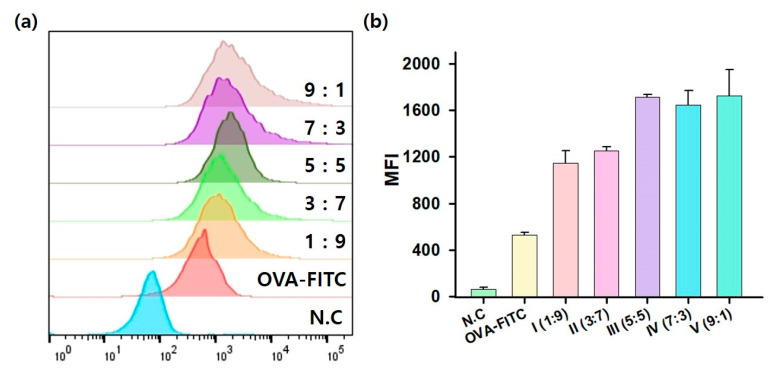
Comparative analysis of cell fluorescence uptake conducted using a flow cytometer. RAW 264.7 cells were exposed to OVA–FITC-only and OVA–FITC-encapsulated ChargeSomes for 6 h. (**a**) Histograms of the samples (ratio of mPEG-b-PLL to mPEG-b-PLL-SA). (**b**) Median fluorescence intensity (MFI) values for the samples. Data are presented as means ± S.D. (n = 3).

Cell uptake by specific cells, particularly antigen-presenting cells (APCs), is crucial for mRNA delivery systems such as ChargeSomes. APCs increase mRNA expression rate, leading to vaccine effects [51]. To evaluate the efficiency of ChargeSomes, in vitro evaluation was performed using CLSM and fluorescence-activated single-cell sorting. OVA conjugated with the fluorescent molecule FITC (FITC–OVA) was used as a model antigen as an optical marker for tracking the cell uptake of ChargeSomes [45]. As shown in Figure 3, OVA–FITC-encapsulated ChargeSomes were added to RAW 264.7 cells. As indicated by the green fluorescence, OVA–FITC was taken up by the cells. By employing CLSM, we verified that OVA–FITC-encapsulated ChargeSomes exhibited superior cell uptake compared to that of OVA–FITC alone. Across all (mPEG-b-PLL: mPEG-b-PLL-SA) ratios, there was a substantial increase in the green fluorescence intensity of ChargeSomes when encapsulated in OVA–FITC, underscoring the effectiveness of the carrier [52]. These results were further corroborated by the flow cytometry results shown in Figure 4. The fluorescence levels of OVA–FITC-encapsulated ChargeSomes consistently exceeded those of the OVA–FITC control group. These results demonstrate that ChargeSomes enhance cellular uptake, highlighting their potential as effective mRNA delivery vehicles. In particular, the surface charge of ChargeSomes plays a critical role, as a higher surface charge increases the efficiency of cellular uptake through interaction with the cell surface.

### 3.5. Endosomal Escape Dynamics of ChargeSomes

**Figure 5:**
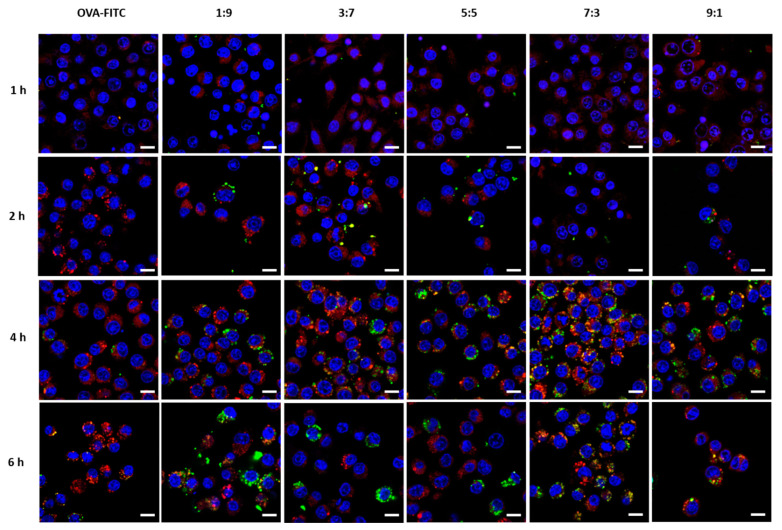
Cell uptake and endosomal escape of ChargeSomes. Negative control and OVA–FITC-encapsulated ChargeSome samples were administered and cultured for 1, 2, 4, and 6 h. Subsequently, endosomal escape of the particles was confirmed by employing a confocal laser-scanning microscope. RAW 264.7 cells were incubated with OVA–FITC-encapsulated ChargeSomes (green), lysosomes, and nuclei were stained using Lysotracker-DND-99 (red) and Hoechst 33342 (blue), respectively. Scale bars represent 10 um.

To determine the cell interactions of ChargeSomes, we analyzed the endosomal escape dynamics after cell internalization [53]. Endosome escape is the most important factor in any mRNA delivery system, as mRNAs must exit the endosomes and reach the cytoplasm for translation into proteins [31]. We used CLSM to analyze the intracellular endosome escape of OVA–FITC-encapsulated ChargeSomes over time. Figure 5 shows the cells incubated with ChargeSomes over a temporal gradient (1, 2, 4, and 6 h) [54]. The red fluorescence Lysotracker DND-99 was used to demarcate endosome boundaries. By observing the overlapping fluorescence (yellow areas), we can infer the colocalization of green OVA–FITC and red endosomes, suggesting their simultaneous presence within the same endosomal structure. Initial observations at 1 h showed no green OVA–FITC fluorescence, indicating that no ChargeSomes endocytosis had occurred at this point. However, after 2 h, colocalized yellow fluorescence indicated that the ChargeSomes were indeed within the endosomes. After 4 h, we observed that colocalization (yellow area) had disappeared. Green OVA–FITC fluorescence was mostly observed, indicating that ChargeSomes successfully escaped the endosomes and dispersed into the cytoplasm. Temporal observation of this endosomal escape provides two important pieces of information. First, the rapid transition from cell uptake to endosomal escape in just 6 h indicated the efficiency of ChargeSomes. This characteristic not only enhances their mRNA translation potential but also reduces the vulnerability period during which mRNAs can be degraded within endosomes. Second, the observed escape patterns provide a mechanistic characterization of endosomal behavior [55]. This endosomal escape is facilitated by the proton sponge effect of mPEG-b-PLL. When the endosomal environment becomes acidic, protons and water are drawn into the endosomes, causing them to swell and eventually rupture. As a result, ChargeSome cargo is released into the cytoplasm. These results regarding the dynamics of endosome escape indicate the potential of ChargeSomes as an mRNA delivery system. Combined with previous results on cell uptake and viability, these results confirmed the potential of ChargeSomes as an mRNA-based vaccine delivery platform.

### 3.6. EGFP mRNA Transfection of ChargeSomes In Vitro

**Figure 6:**
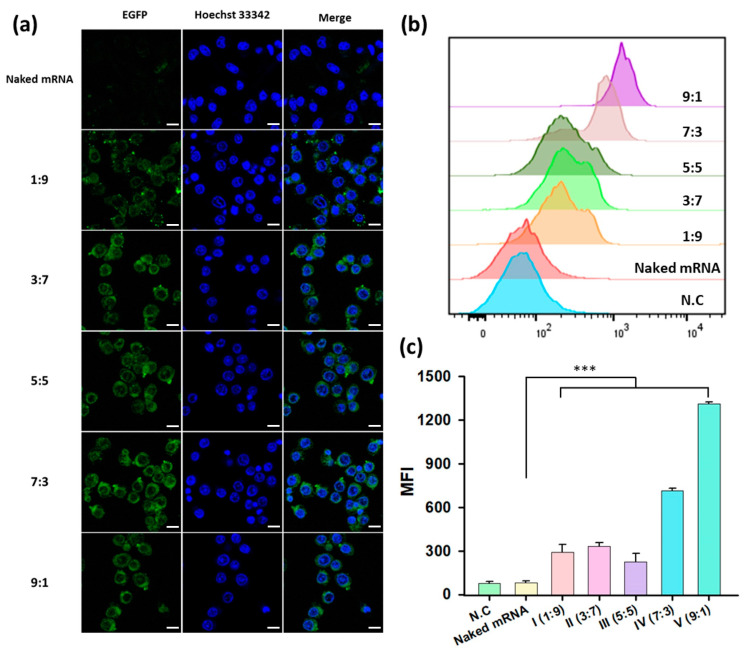
Cell uptake and EGFP mRNA expression. (**a**) RAW 264.7 cells were treated with EGFP mRNA-encapsulated ChargeSomes and naked mRNA separately, followed by CLSM analysis after 24 h of incubation. The efficiency of antigen cell absorption was determined by flow cytometry to assess EGFP expression. RAW 264.7 cells were expressed with EGFP mRNA-encapsulated ChargeSomes (green) and nuclei were stained using Hoechst 33342 (blue), respectively. (**b**) Histogram representing the negative control (N.C) and each sample. (**c**) Median fluorescence intensity (MFI) for both the negative control and each sample. Student’s *t*-test and one-way ANOVA were used for statistical analysis. Data are presented as means ± S.D. (n = 3; *** *p* < 0.001). Scale bars represent 10 um.

To investigate the application of foreign genes on RAW 264.7 cells using mRNA encapsulated in ChargeSomes, we used a model mRNA expressing EGFP. EGFP mRNA encapsulation was confirmed by gel electrophoresis, and the encapsulated mRNAs were well protected in the presence of PBS, RNase, and FBS (Figure S7) [46,47,56]. A certain amount of EGFP mRNA was used to generate mRNA-encapsulated ChargeSomes for each ChargeSome ratio (1:9, 3:7, 5:5, 7:3, and 9:1). RAW 264.7 cells were treated with ChargeSomes and naked mRNA and co-cultured for 24 h, after which mRNA expression was evaluated using CLSM [56]. As shown in the CLSM images in Figure 6a, it was evident that no EGFP was expressed in cells treated with naked mRNA. This absence suggests that unencapsulated mRNA was likely degraded during endocytosis or was not internalized by the cells, resulting in no cytoplasmic mRNA expression. In contrast, significant EGFP expression was observed in cells treated with EGFP mRNA encapsulated in ChargeSomes at various ratios (1:9, 3:7, 5:5, 7:3, and 9:1). Notably, the highest EGFP expression was observed at a 9:1 ChargeSome ratio, as shown in the CLSM images. Quantitative evaluation was performed using flow cytometry (Figure 6b,c). By comparing EGFP expression, it was clear that mRNA delivery via ChargeSomes significantly improved the efficiency of EGFP expression compared to that using naked mRNA alone, as shown in Figure S8. These results indicated that EGFP can effectively evade endosomes and successfully and efficiently deliver mRNA to the cytoplasm. Interestingly, the efficiency of EGFP expression was distinctly higher when using a 9:1 ChargeSome ratio than when using other irradiation ratios. This behavior is consistent with the observed fluorescence intensity. The higher expression efficiency was attributed to the inherent properties of the polymer. These results highlight the potential of ChargeSomes as a vaccine delivery vehicle capable of efficiently transporting and transducing mRNA.

## 4. Conclusions

Messenger RNA (mRNA) therapies have attracted considerable interest owing to their potential for custom protein expression and therapeutic versatility. To overcome challenges such as instability and degradation, we developed ChargeSomes as a platform for vaccine delivery vehicles. ChargeSomes, which are derived from the electrostatic interaction between the cationic polymer mPEG-b-PLL and the anionic polymer mPEG-PLL-SA, exhibit distinctive properties that improve mRNA delivery. The effective synthesis of the copolymers was validated by ^1^H NMR and FT-IR spectroscopies. The physicochemical properties of these ChargeSomes were confirmed using TEM and DLS analyses. At a mPEG-b-PLL to mPEG-b-PLL-SA ratio of 9:1, ChargeSomes exhibited improved cell uptake, endosome escape, and mRNA delivery efficiency in OVA–FITC uptake and EGFP mRNA transfection. ChargeSomes demonstrated low cytotoxicity in various structures, confirming their potential for therapeutic applications. Specifically, their pH sensitivity in acidic environments, similar to that of endosomes, results in an increased capacity for mRNA delivery. In addition, ChargeSomes are more stable at neutral pH, and their sophisticated design shields mRNA from RNase degradation, while maximizing endosome escape via the proton sponge effect. This not only shields mRNA but also facilitates its efficient delivery to the cytoplasm of target cells. The unique physicochemical properties of ChargeSomes, combined with their cell uptake, endosome avoidance, and mRNA protection, provide a stable mRNA delivery solution and show significant potential for advancing the development of mRNA-based therapeutics and vaccines.

## Data Availability

Data are contained within the article and Supplementary Materials.

## Supplementary Materials

The following supporting information can be downloaded at https://www.mdpi.com/article/10.3390/pharmaceutics15122781/s1. Figure S1. FT-IR spectra of Lys(Z)-NCA, mPEG-b-PLL(Z), mPEG-b-PLL, mPEG-b-PLL-SA. Figure S2. ^1^H NMR spectra of copolymers. (a) Lys(Z)-NCA (DMSO-d_6_; 25 °C) (b) mPEG-b-PLL(Z) (DMSO-d_6_; 25 °C), (c) mPEG-b-PLL (D_2_O; 25 °C), (d) mPEG-b-PLL-SA (D_2_O; 25 °C). Figure S3. ChargeSomes (mPEG-b-PLL: mPEG-b-PLL-g-SA = 0:10, and 10:0) were analyzed using negative staining by transmission electron microscopy (TEM). The scale bar indicates 200 nm. Figure S4. ChargeSomes observed by scanning electron microscopy (SEM). The scale bar represents 100 nm. Figure S5. (a) For stability assessment, the size distribution of ChargeSomes was monitored in PBS (pH 7.4) over a span of 6 weeks. (b) To verify pH responsiveness, alterations in the size of ChargeSomes were observed in PBS (pH 5.0) over a period ranging from 0 h to 2 days. Figure S6. Cell uptake and endosomal escape of ChargeSomes. Unmerged images depicting the endosomal escape of ChargeSomes at various time points (1, 2, 4, and 6 h) for both negative control and OVA–FITC-encapsulated ChargeSome samples using a confocal laser-scanning microscope. Figure S7. Electrophoretic analysis of the mRNA-encapsulated ChargeSomes was applied to an agarose gel and subjected to electrophoresis. Retardation of the mRNA was visualized using Neogreen. (a) Gel electrophoresis was employed to ascertain the mRNA-encapsulation rate based on ChargeSome concentration and to identify the concentration point at which free mRNA bands were no longer observable. (b) Stability of naked mRNA alone and mRNA-encapsulated ChargeSomes in different solvents (PBS, FBS, RNase) was assessed. Figure S8. mRNA expression positivity efficiency of RAW 264.7 cells cultured 24 h after treatment with either EGFP mRNA alone or EGFP mRNA-encapsulated ChargeSomes. Figure S9. Comparative analysis of cell fluorescence uptake. RAW 264.7 cells were exposed to OVA–FITC-encapsulated ChargeSome (9:1) and lipofectamine for 6 h. Figure S10. Size distribution of ChargeSomes (0:10, 10:0) determined by dynamic light scattering. Table S1. Cell viability of RAW 264.7 cells at 12 h, and 24 h post-treatment with ChargeSomes (9:1) or lipofectamine was evaluated using the EZ-Cytox assay (n = 4).
