# Supplementary material for: Charge-Complementary Polymersomes for Enhanced mRNA Delivery
Source: Pharmaceutics. 2023 Dec 15;15(12):2781. doi: 10.3390/pharmaceutics15122781 (PMC10748362; doi:10.3390/pharmaceutics15122781)
Supplement: Supplementary file 1 [file pharmaceutics-15-02781-s001.zip › pharmaceutics-2747610-supplementary.pdf]

Supporting Information

Title: Charge-Complementary Polymersomes for Enhanced mRNA Delivery

HakSeon Kim[1, 2,†], Yu-Rim Ahn[1, 2,†], Minse Kim[1, 2], Jaewon Choi[1, 2], SoJin Shin[1, 2] and Hyun-Ouk Kim [1, 2]*

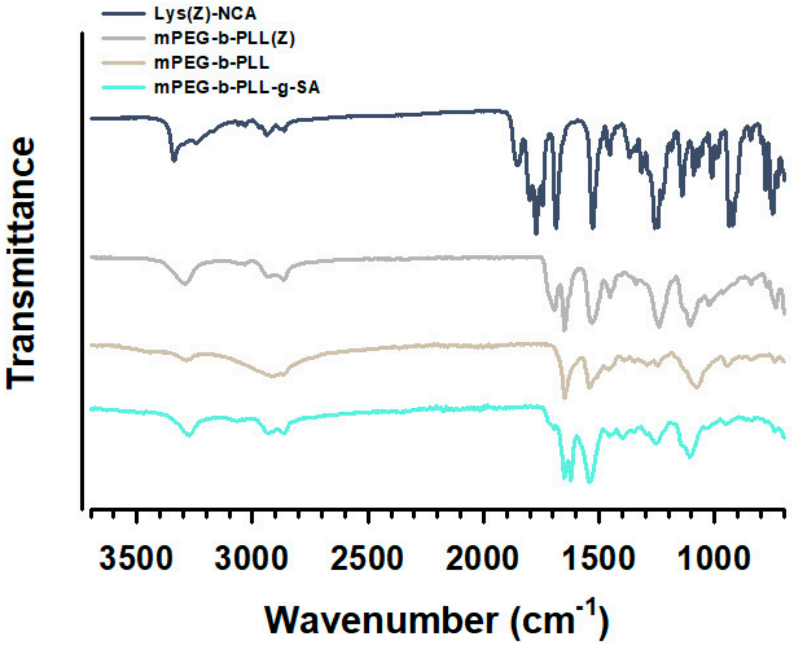

Figure S1. FT-IR spectra of Lys(Z)-NCA, mPEG-b-PLL(Z), mPEG-b-PLL, mPEG-b-PLL-SA.

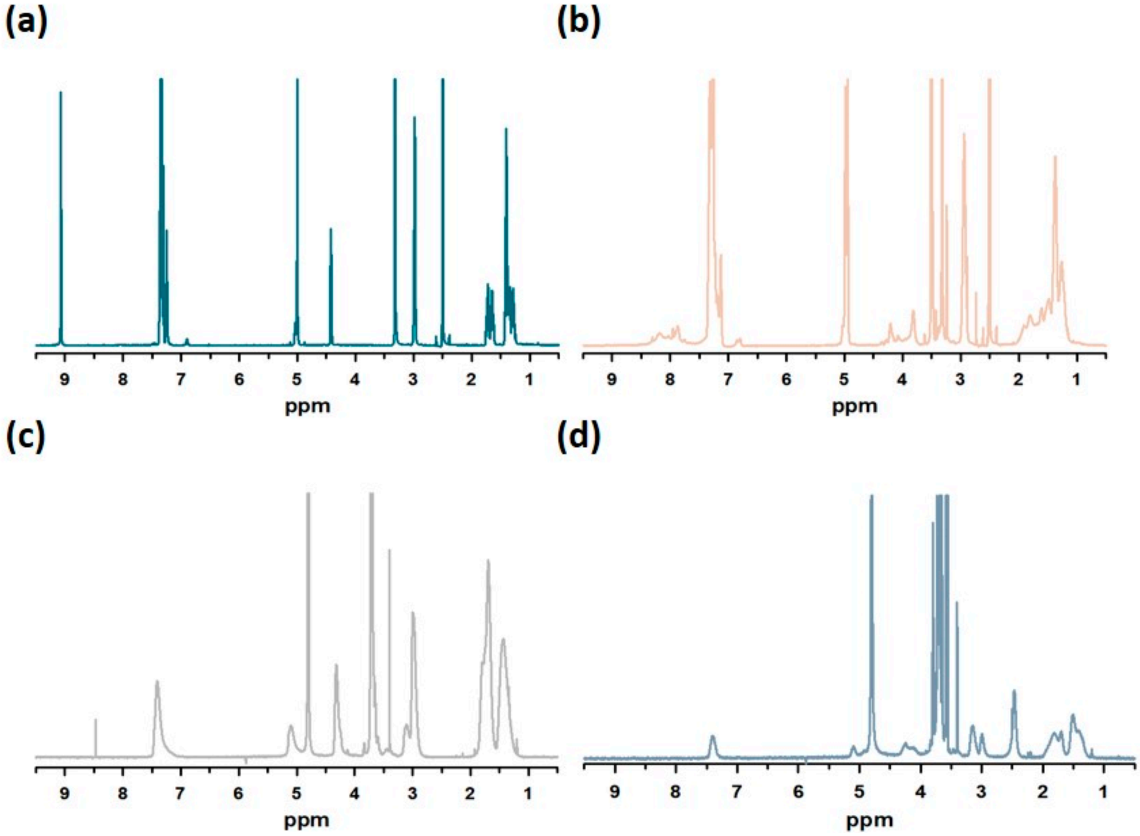

Figure S2. ^{1}**H NMR spectrum of copolymers. (a) Lys(Z)-NCA (DMSO-d_6; 25 °C) (b) mPEG-b-PLL(Z) (DMSO-d_6; 25 °C), (c) mPEG-b-PLL (D_2O; 25 °C), (d) mPEG-b-PLL-SA (D_2O; 25 °C).**

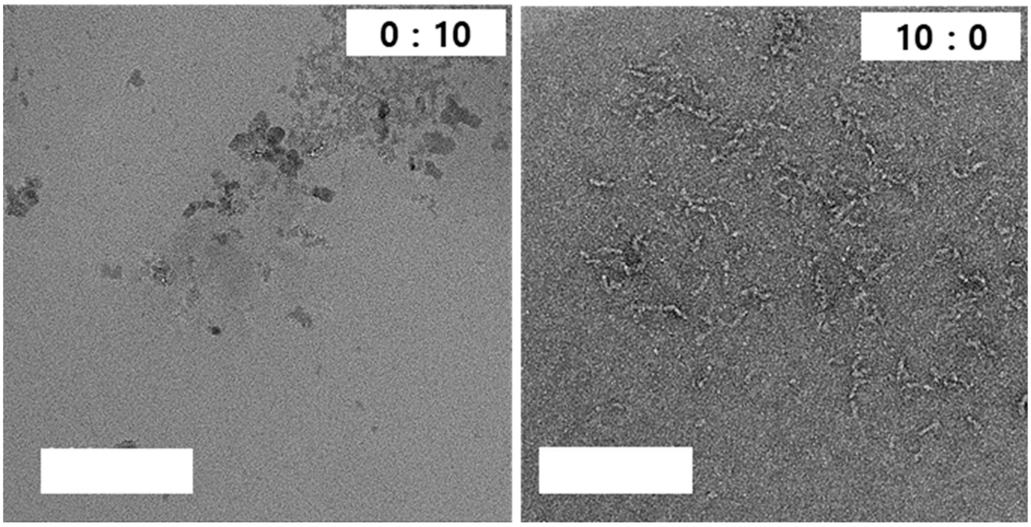

Figure S3. ChargeSome (mPEG-b-PLL: mPEG-b-PLL-g-SA = 0:10, and 10:0) were analyzed using negative staining by transmission electron microscopy (TEM). The scale bar indicates 200 nm.

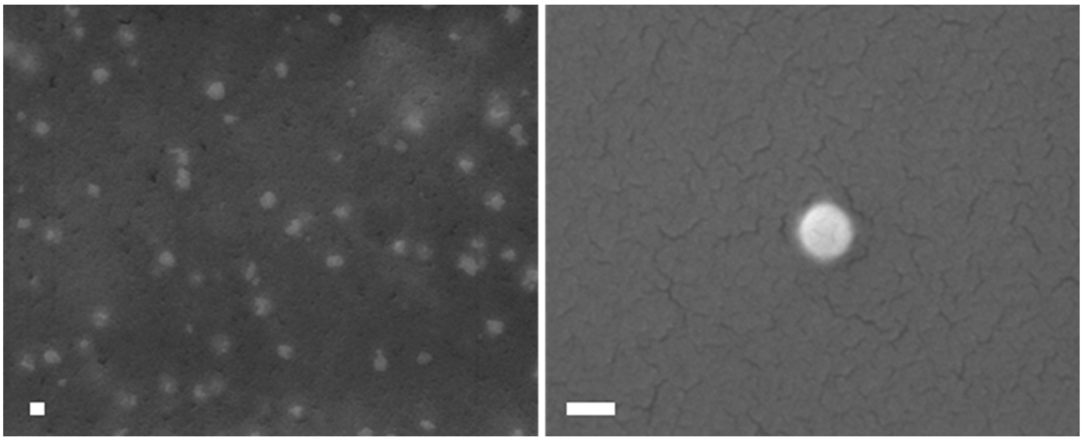

Figure S4. ChargeSome observed by scanning electron microscopy (SEM). The scale bar represents 100 nm.

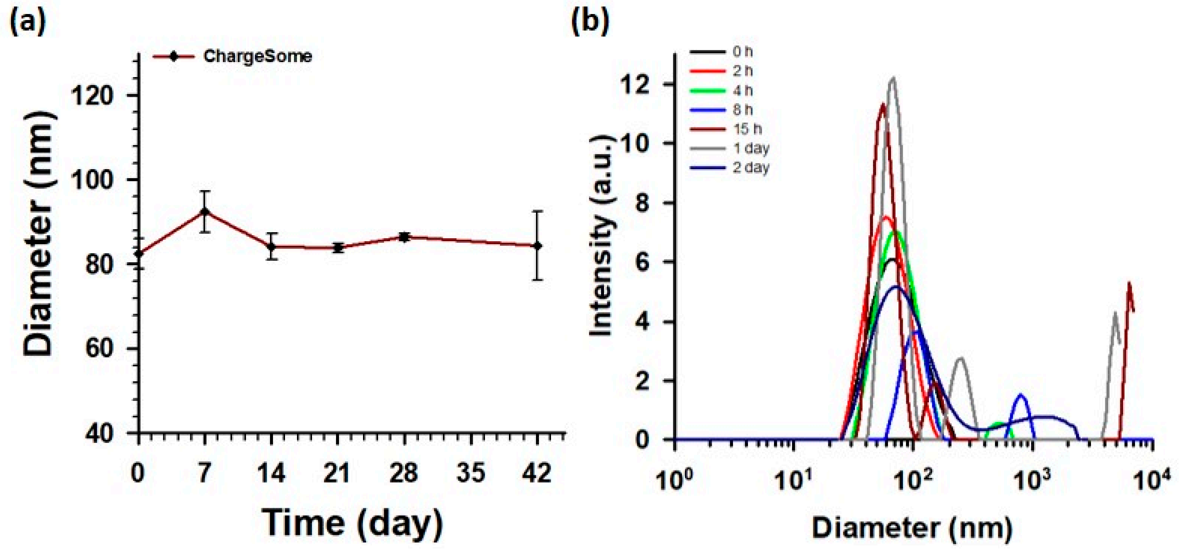

Figure S5. (a) For stability assessment, the size distribution of ChargeSome was monitored in PBS (pH 7.4) over a span of 6 weeks. (b) To verify pH responsiveness, alterations in the size of ChargeSome were observed in PBS (pH 5.0) over a period ranging from 0 hours to 2 days.

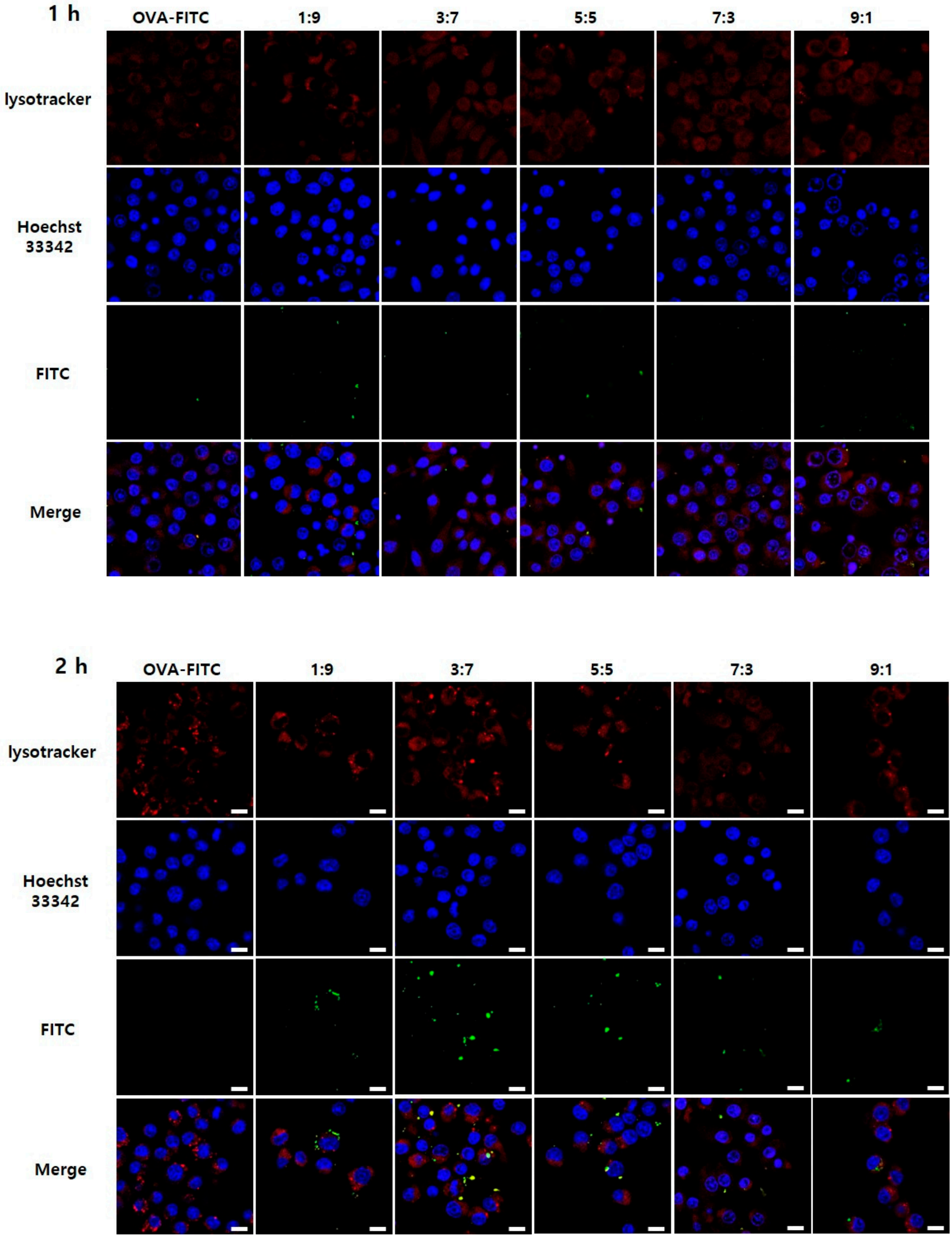
1 h
OVA-FITC
1:9
3:7
5:5
7:3
9:1
lysotracker
Hoechst 33342
FITC
Merge
2 h
OVA-FITC
1:9
3:7
5:5
7:3
9:1
lysotracker
Hoechst 33342
FITC
Merge

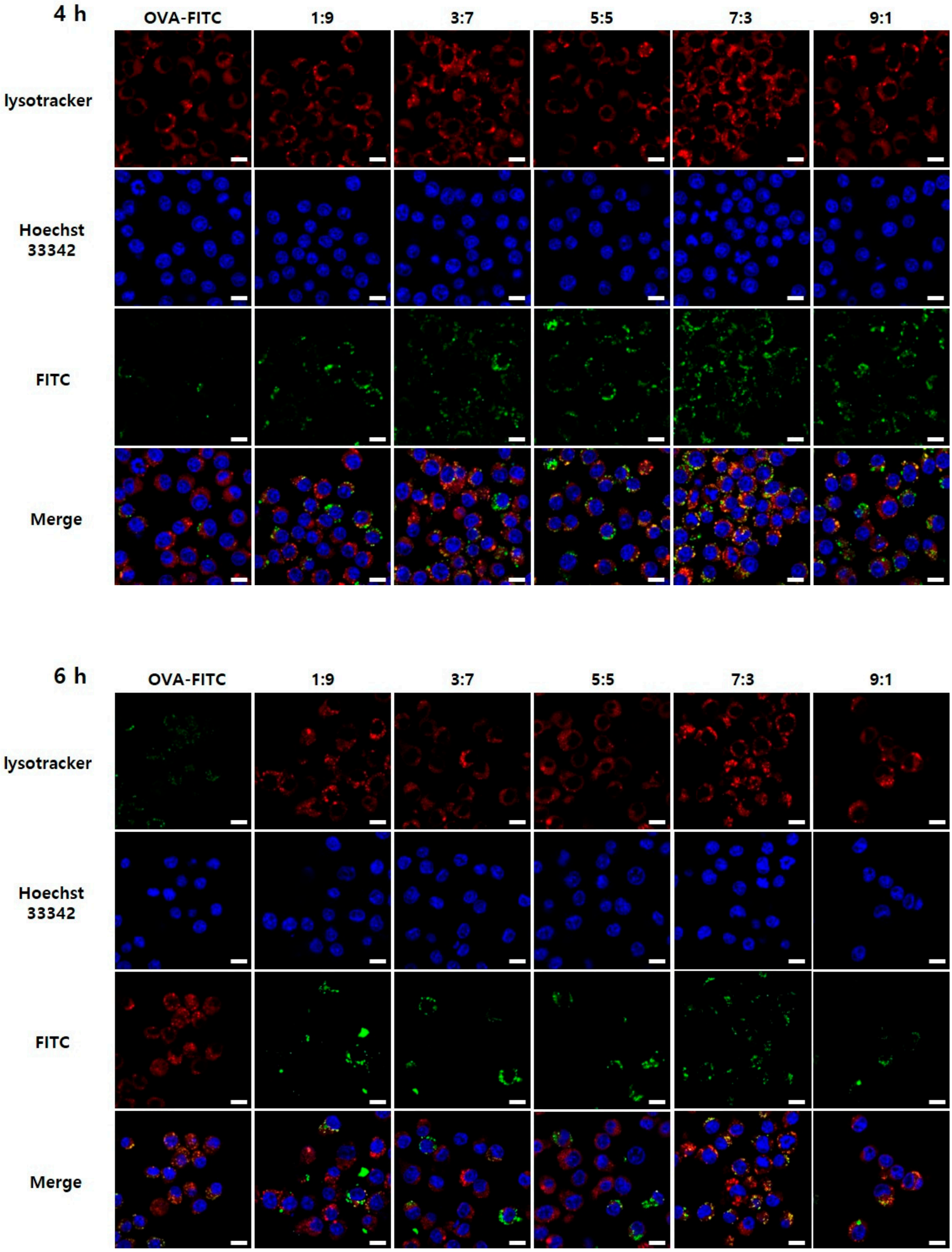

Figure S6. Cell Uptake and Endosomal Escape of ChargeSome. Unmerged images depicting the endosomal escape of ChargeSome at various time points (1, 2, 4, and 6 h) for both negative control and OVA-FITC-encapsulated ChargeSome samples by using a confocal laser scanning microscope.

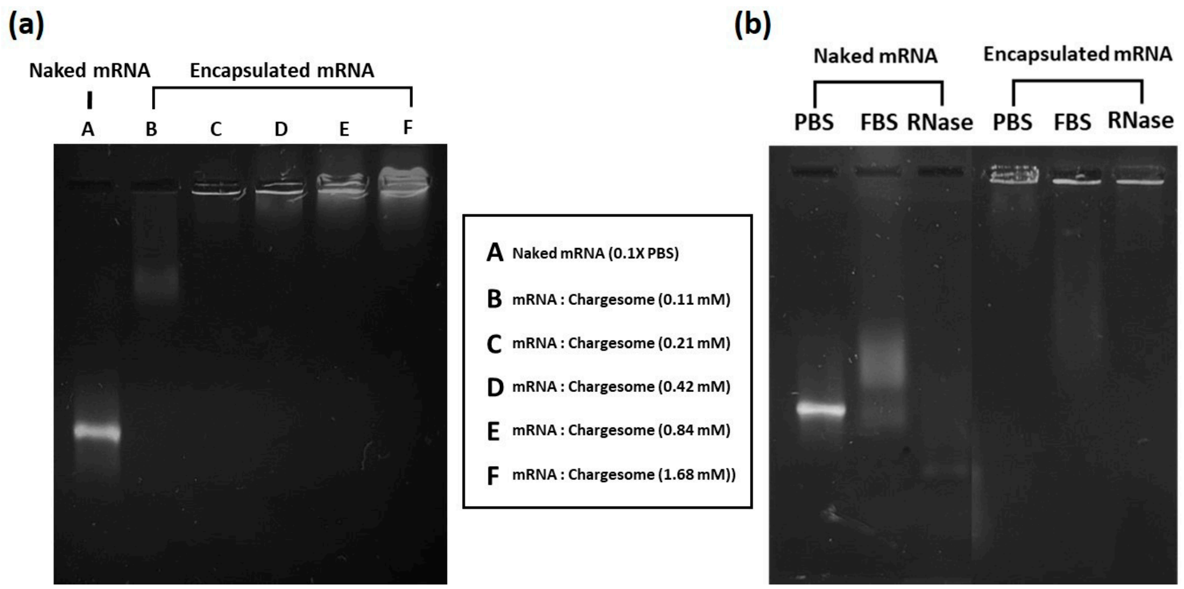

Figure S7. Electrophoretic analysis of the mRNA encapsulated chargeSome was applied to an agarose gel and subjected to electrophoresis. Retardation of the mRNA was visualized using Neogreen. (a) Gel electrophoresis was employed to ascertain the mRNA encapsulation rate based on ChargeSome concentration and to identify the concentration point at which free mRNA bands were no longer observable. (b) stability of naked mRNA alone and mRNA-encapsulated ChargeSome in different solvents (PBS, FBS, RNase) was assessed.

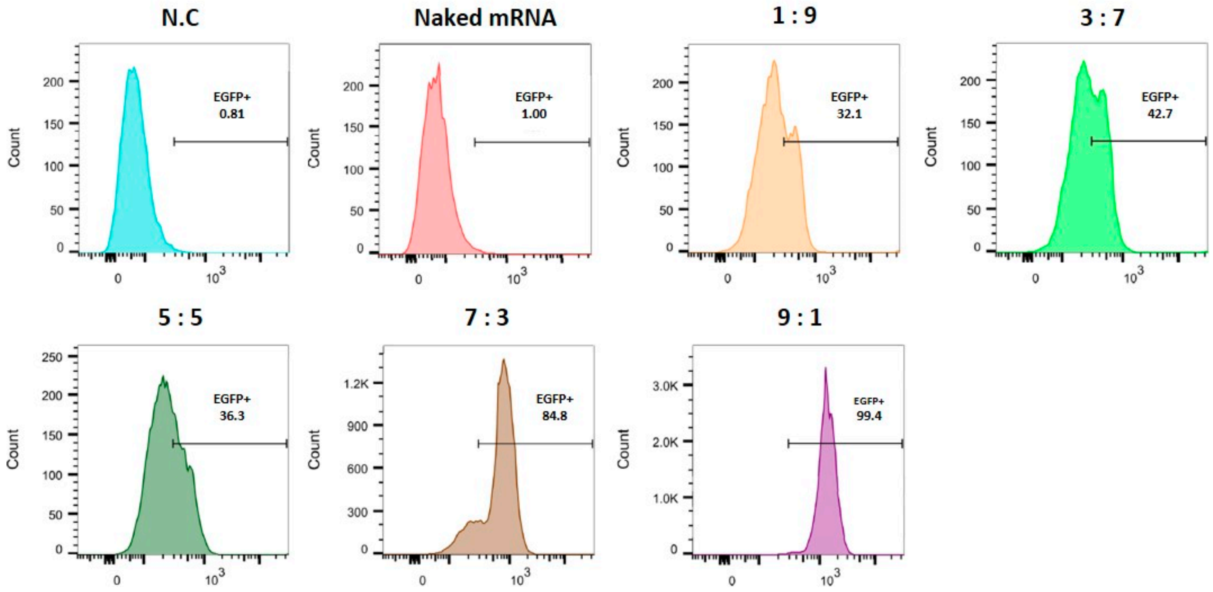

Figure S8. mRNA expression positivity efficiency of RAW 264.7 cells cultured 24 hours after treatment with either EGFP mRNA alone or EGFP mRNA encapsulated ChargeSome.

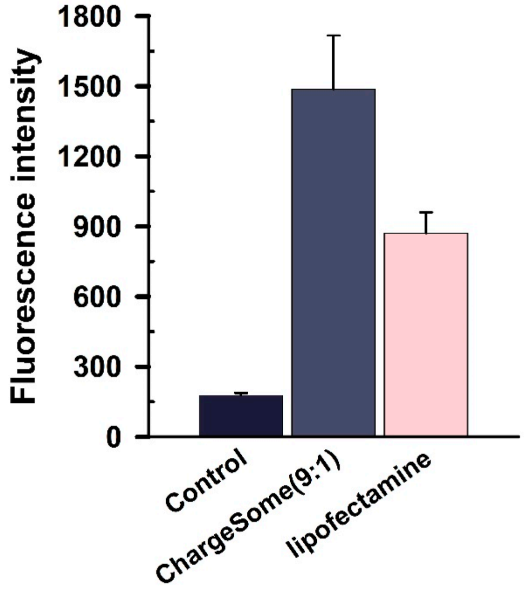

Figure S9. Comparative analysis of cell fluorescence uptake. RAW 264.7 cells were exposed to OVA-FITC-encapsulated ChargeSome (9:1) and lipofectamine for 6 h.

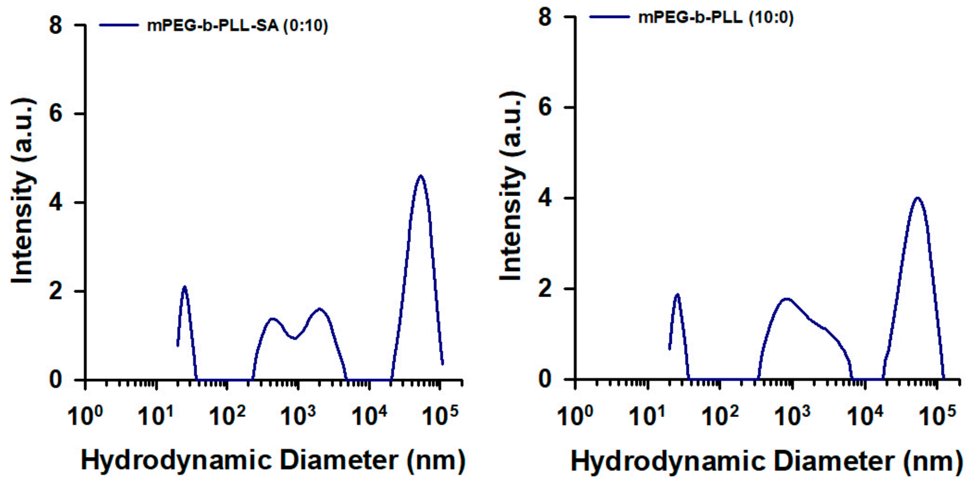

Figure S10. Size distribution of ChargeSomes (0:10,10:0) determined by dynamic light scattering.

Table S1. Cell viability of Raw 264.7 cells at 12 h, and 24 h post-treatment with ChargeSome (9:1) or lipofectamine was evaluated using the EZ-Cytox assay (n=4).

Incubation time (h)	Cell viability (%)	
	ChargeSome (9:1)	lipofectamine
12	97.56 ± 1.62	91.74 ± 1.30
24	94.74 ± 2.05	88.67 ± 1.44
